# High Bifurcation of the Common Carotid Artery with Pentafurcation of the External Carotid Artery: Case Series with Review of Literature

**DOI:** 10.15388/Amed.2025.32.2.12

**Published:** 2025-12-30

**Authors:** Aarthi Manokaran, Manisha R Gaikwad, Baskar A, Murugan Gopalakrishnan, Praveen Kumar Ravi

**Affiliations:** 1 Department of Anatomy, All India Institute of Medical Sciences, Bhubaneswar, Odisha, India; 2 Department of Anatomy, All India Institute of Medical Sciences, Bhubaneswar, Odisha, India; 3 Department of Radiodiagnosis, Sree Balaji Medical College and Hospital, Chennai, Tamil Nadu, India; 4 Department of Radiodiagnosis, Sree Balaji Medical College and Hospital, Chennai, Tamil Nadu, India; 5 Department of Anatomy, All India Institute of Medical Sciences, Bhubaneswar, Odisha, India

**Keywords:** high carotid bifurcation, carotid surgery, preoperative imaging, miego arterijos šakojimasis, miego arterijos operacija, ikioperacinis vaizdinimas

## Abstract

The bifurcation of the *Common Carotid Artery* (CCA) is a pivotal anatomical landmark in head and neck surgeries, particularly affecting procedures such as carotid endarterectomy. Typically occurring around the C3-C4 intervertebral disc, variations in its location can complicate surgical access and pose risks of nerve injury. In this case report, three rare bilateral high bifurcations of the CCA at the C2 and C2/3 vertebrae levels are documented. Additionally, an unusual pentafurcation of the *External Carotid Artery* (ECA) and bilateral superior thyroid artery are observed, arising directly from the CCA. The high bifurcation presents challenges due to its proximity to nerves such as the hypoglossal nerve, potentially leading to complications such as dysphagia or speech impairment. Furthermore, it complicates procedures like carotid endarterectomy, necessitating additional techniques for adequate exposure and increasing risks of *Internal Carotid Artery* (ICA) thrombosis. Detailed preoperative imaging before surgery is important for effective planning and minimizing the surgical risk. Anatomical variation, such as the vascular structure, can significantly affect surgical and interventional outcomes. Identifying these differences through meticulous evaluation enables surgeons to anticipate potential challenges, reduce complications, and enhance patient outcomes.

## Abstract (it)

Miego arterijos (CCA) šakojimasis yra svarbus anatominis orientyras galvos ir kaklo operacijoms, ypač atliekant tokias procedūras kaip miego arterijos endarterektomija. Paprastai šis šakojimasis būna aplink C3-C4 tarpslankstelinį diską, tačiau jo vietos variacijos gali apsunkinti chirurginę prieigą ir didinti nervų pažeidimo riziką. Šiame atvejo aprašyme dokumentuojami trys reti abipusiai didelės CCA bifurkacijos atvejai C2 ir C2/3 slankstelių lygiu. Be to, pastebimas neįprastas išorinės miego arterijos (ECA) penkiakampis šakojimasis ir abipusė viršutinė skydliaukės arterija, kylanti tiesiai iš CCA. Didelė bifurkacija kelia sunkumų dėl artumo nervams, pvz., hipoglosaliniam nervui, ir gali sukelti komplikacijų, pvz., disfagiją ar kalbos sutrikimus. Be to, ji apsunkina tokias procedūras, kaip antai karotidinės endarterektomijos, tada reikia papildomų technikų ir didina vidinės karotidinės arterijos (ICA) trombozės riziką. Išsamus ikioperacinis vaizdinis tyrimas yra svarbus veiksmingam operacijos planavimui ir chirurginei rizikai sumažinti. Anatomijos skirtumai, pvz., kraujagyslių struktūra, gali turėti didelę įtaką chirurginių ir intervencinių procedūrų rezultatams. Šių nustatytų skirtumų atidus įvertinimas leidžia chirurgams numatyti galimas problemas, sumažinti komplikacijų riziką ir pagerinti pacientų gydymo rezultatus.

## Introduction

The bifurcation of the *Common Carotid Artery* (CCA) is a pivotal anatomical landmark in head and neck surgery, particularly in vascular procedures, tumor resections, and reconstructive surgeries. Typically, carotid bifurcation occurs at the C3-C4 intervertebral disk level or the superior border of the thyroid cartilage when the head is in the Frankfurt plane; the bifurcation gives rise to the *Internal Carotid Artery* (ICA) and *External Carotid Artery* (ECA) [1]. However, variations in its location and branching pattern have been extensively documented, located anywhere between the C3-C4 intervertebral and C5/6 intervertebral disk [2]. When the variation is higher than the upper border of C3, it is difficult to access the carotid bifurcation and ICA intraoperatively during carotid endarterectomy or stenting. The nerves, including hypoglossal and marginal mandibular, will be closely related to high carotid bifurcation, leading to nerve injury during surgical exploration [3].

The external carotid artery supplies numerous structures within the head and neck, giving rise to branches such as the *Superior Thyroid Artery* (STA) and the *Ascending Pharyngeal Artery* (APA). Notably, the ascending pharyngeal artery stands out as the sole medial branch, traversing deep to the ICA, while the STA originates from the anterior surface of the ECA near the hyoid bone [4]. A comprehensive understanding of these branches is indispensable for surgical interventions, particularly in cases involving vascular anomalies. In this case report, we present a rare anatomical variation of the high bifurcation of CCA and variation in the ECA branching pattern, emphasizing its clinical significance and potential implications for surgical planning. A thorough comprehension of such variations can facilitate enhanced surgical precision and mitigate the risk of vascular or neural complications.

## Case Report

**Figure 1:**
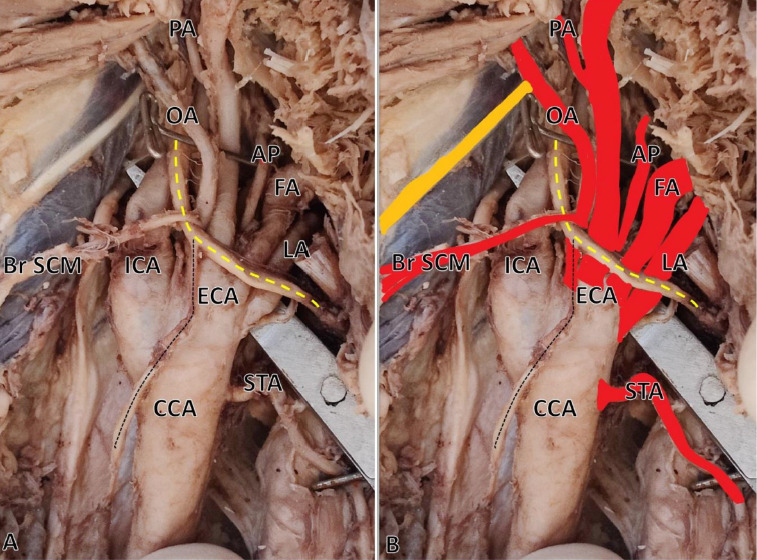
Photograph (A) and colored photograph (B) of right-sided high carotid bifurcation and pentafurcation of external carotid artery (ECA); superior thyroid artery (STA) directly arises from the common carotid artery (CCA). Just below the looping of the hypoglossal nerve (the yellow dotted line), ECA branches into the lingual (LA), facial (FA), ascending pharyngeal (AP), and occipital artery (OA). The posterior auricular artery (PA) arises from the common trunk that branches into superficial temporal and maxillary arteries; the branch to sternocleidomastoid (Br SCM) artery arises from the OA, which hocks around the hypoglossal nerve; the black dotted line refers to the superior root of the ansa cervicalis; the solid yellow line indicates the spinal accessory nerve.

**Figure 2:**
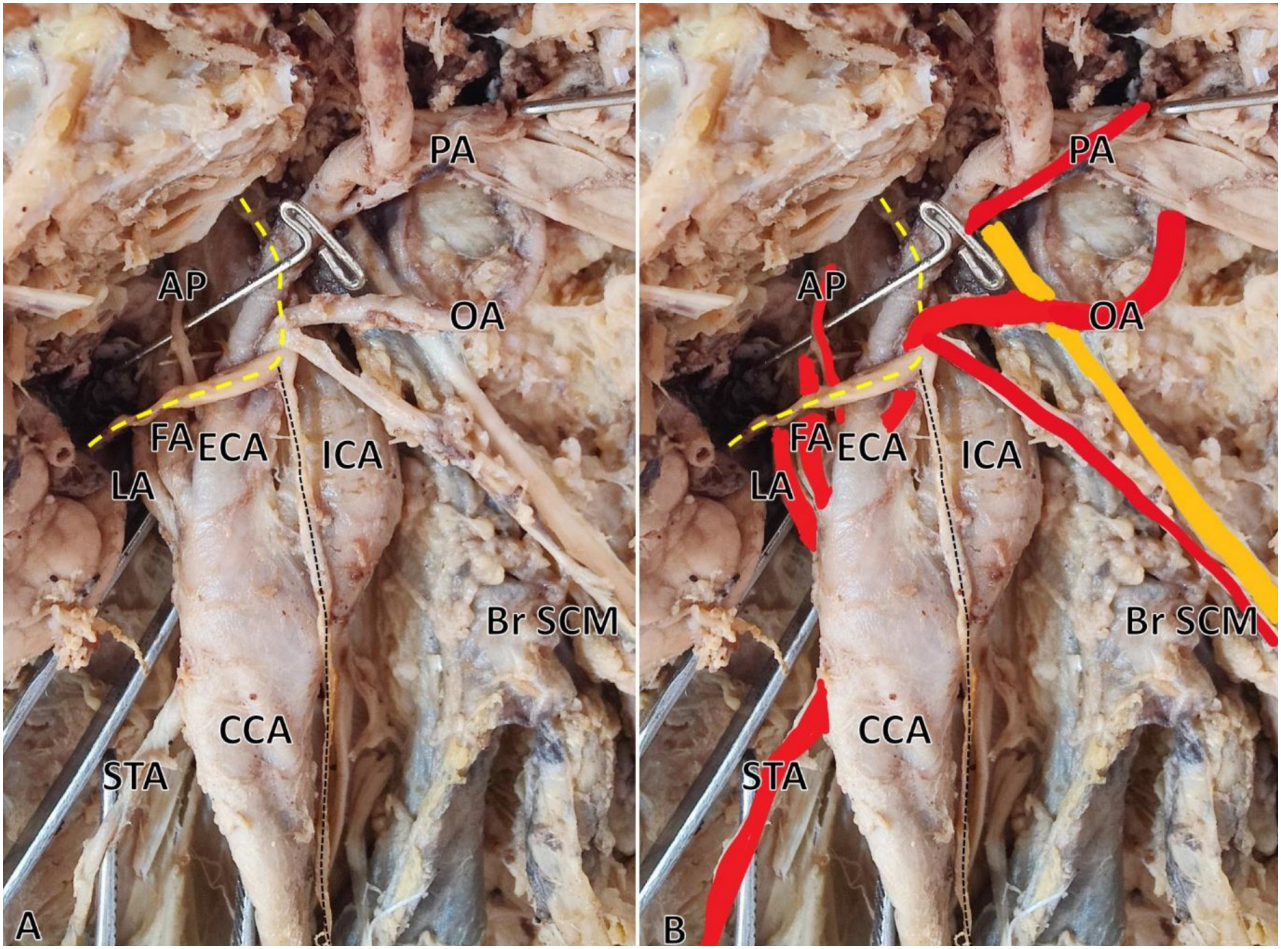
Photograph (A) and colored photograph (B) of high carotid bifurcation and its branching pattern of the left external carotid artery (ECA); superior thyroid artery (STA) directly arises from the common carotid artery (CCA). Just below the looping of the hypoglossal nerve (the yellow dotted line), ECA branches into the lingual (LA), facial (FA) and ascending pharyngeal (AP); the occipital artery (OA), posterior auricular artery (PA) and other branches arise from the common trunk; the branch to the sternocleidomastoid (Br SCM) artery arises from the OA, which hocks around the hypoglossal nerve; the black dotted line refers to the superior root of the ansa cervicalis; the solid yellow line indicates the spinal accessory nerve.

***Case 1:*** During the routine cadaveric dissection conducted for undergraduate medical students at the Department of Anatomy on formalin-fixed embalmed cadavers, a bilateral high bifurcation of the *Common Carotid Artery* (CCA) was observed in a 55-year-old male cadaver. The dissection was performed in accordance with the Cunningham’s Manual of Practical Anatomy, 16^th^ edition. Additionally, a variation in the branching pattern was noted in the ECA. CCA bifurcation was observed just above the level of the angle of the mandible (C2 vertebrae) against the usual bifurcation at the level of the C3-C4 vertebrae. Bilaterally, the superior thyroid artery arises from the CCA. On the right side, at the carotid sinus, the ECA gave off five branches (pentafurcation) immediately, including the ascending pharyngeal, lingual, facial, occipital, and a common trunk for posterior auricular, superficial temporal, and maxillary arteries, which all arose from a single point, which is unusual (Fig. 1). On the left side, at the level of the carotid sinus, the ECA gave off the ascending pharyngeal, lingual, and facial arteries, and the common trunk provided the occipital and other branches (Fig. 2). The muscular branch from the occipital artery looped around the hypoglossal nerve to supply the sternocleidomastoid muscle bilaterally. Bilaterally, a curvilinear looping of the common trunk of the posterior auricular, superficial temporal, and maxillary arteries was observed prior to their entry into the substance of the parotid gland.

**Figure 3:**
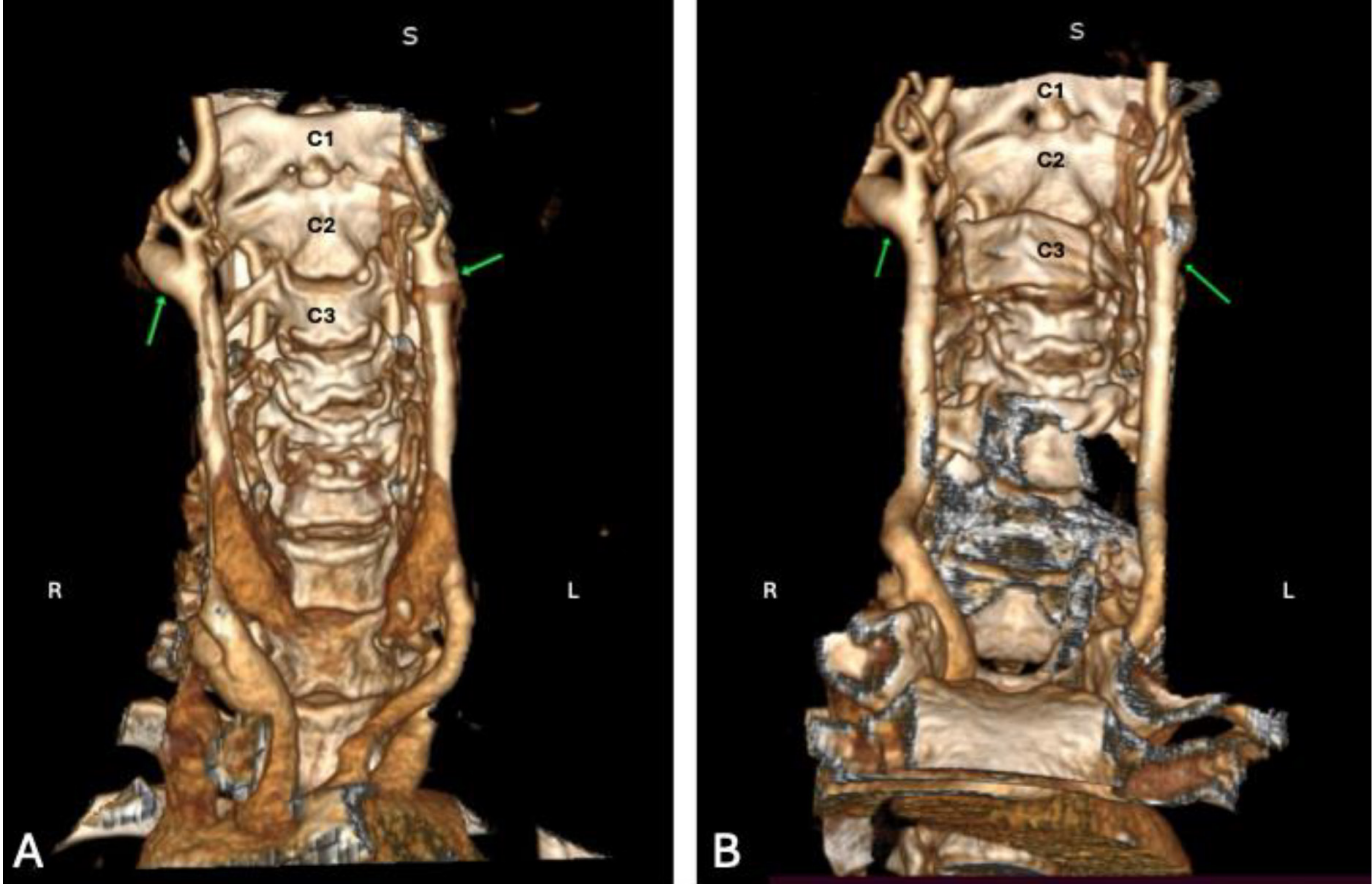
3D Rendered Image of Computed Tomography Head and Neck Angiography (CT Angiography). This 3D-rendered image depicts two distinct cases (A and B) of bilateral high carotid bifurcation (the green arrow). In both instances, the right-sided bifurcation occurs at the level of the C2 vertebra, while the left-sided bifurcation is situated between the C2 and C3 vertebrae. Note. S – Superior; R – Right side; L – Left side.

***Case 2:*** A 70-year-old male presented with complaints of vertigo and was referred for *Computed Tomography* (CT) angiography of the head and neck to assess for possible vascular causes. The CT angiography was performed by using a 64-slice multi-detector CT scanner (*Siemens GO* 64 slice CT scan, Germany). Following the acquisition of non-contrast images, a contrast-enhanced CT angiogram of the head and neck was performed, covering the region from the ascending aorta up to the cranial vault. A non-ionic contrast medium (Iohexol, 350 mg I/mL) was delivered through the anterior cubital vein at an injection rate of 4.0 mL per second. A total of 80 mL was injected, followed by a saline flush. Bolus tracking was applied with the region of interest placed in the *Region Of Interest* (ROI) ascending aorta with a triggering threshold of 150 *Hounsfield Units* (HU). Scan parameters included a tube voltage of 120 kVp and a tube current of 250 mA. The slice thickness was 3 mm, with an interslice gap of 3 mm. Following image acquisition, the scan data were transferred to the workstation for post-processing, including volume rendering, *Multiplanar Reconstruction* (MPR), and *Maximum Intensity Projection* (MIP). The examination revealed middle cerebral artery stenosis on the right side and bilateral higher bifurcation of the carotid artery. On the right side, the bifurcation was situated at the level of C2, while, on the left side, it was located at the intervertebral disc between the C2 and C3 vertebrae (Figure 3A). The branching pattern of the external carotid artery was found to be normal.

***Case 3:*** In this instance, an incidental higher bifurcation of the carotid artery was observed in an 80-year-old female patient who underwent computed tomography head and neck angiography (CT angiography) for complaints of left-sided weakness. The CT angiography was performed as described in Case 2. As depicted in Figure 3B, the patient exhibits bilateral carotid bifurcation at the level of the C2 vertebra on the right side and the intervertebral disc between C2 and C3 on the left side. Notably, there was found no discernible variation in the branching pattern of the external carotid artery.

## Discussion

Carotid bifurcation is typically documented by vertebral levels, although its precise position can vary significantly between studies (Table 1) [5–15]. Generally, the carotid bifurcation is located between the C3/C4 intervertebral disk and the C5/C6 intervertebral disk [1,16]. A bifurcation above the C3/C4 intervertebral disk level, when the head is positioned in the Frankfurt plane, is considered high. However, relying solely on vertebral landmarks to define its position can be impractical during surgeries due to patient positioning and vertebral inaccessibility. To address this, researchers have correlated the carotid bifurcation’s height with anterior neck structures and bony landmarks, such as the mastoid process, the angle of the mandible, the greater cornu and the body of the hyoid bone, the sternal notch, and the sternoclavicular joint. Among these landmarks, the distance between the mastoid process and the carotid bifurcation has demonstrated a significant statistical correlation [17]. Kazantsev et al. categorized carotid bifurcation into three types based on vertebral levels: Type 1 (high): from the upper border of C2 to the C3/4 intervertebral disc; Type 2 (medium): from the upper border of C4 to the lower border of C5; and Type 3 (low): from the C5/6 intervertebral disc to the lower border of C7 [18]. By this classification, all the three present cases exhibit a bilateral Type 1 variation, which is associated with an increased risk of ischemic stroke and complications during carotid endarterectomy and stenting. Another notable variation reported in the literature is the 180-degree carotid bifurcation, which is associated with ICA aneurysms [19]. Consequently, variations in bifurcation and its angulation can lead to changes in turbulence, potentially resulting in aneurysms or predisposing individuals to atherosclerotic plaque due to endothelial injury.

**Table 1:** Common carotid artery bifurcation at different cervical vertebral levels (all values are reported in percentages of the sample size)

Author & Year	Population	Type of Study	Sample Size	C2	C2-C3	C3	C3-C4	C4	C4-C5	C5	C5-C6	C6-C7
**Zümre Ö. et al. (2005)** [9]	Turkey	Cadaver	20	-	-	57.5	-	37.5	-	5	-	-
**Anangure D. et al. (2008)** [5]	Kenya	Cadaver	40	12.5	12.5	38.8	22.5	7.5	-	2.5	-	3.75
**Woldeyes D.H. et al. (2014)** [12]	Ethiopia	Cadaver	13	-	3.85	42.31	15.38	38.46	-	-	-	-
**Radha K. et al. (2014)** [7]	India	Cadaver	67	-	-	11.25	83.75	-	-	5	-	-
**Kurkcuoglu et al. (2015)** [11]	Turkey	Angiography	100	4.9	3.5	38	32	12	-	8	-	3.1
**Padma Badhe et al. (2018)** [6]	India	CT Angio	200	3	4	48	28	16	-	-	-	4
**Jitpun E. et al. (2019)** [10]	Thailand	CT	100	0.5	11.5	19	32	21	13.5	0.5	1.5	-
**Chalise U. et al. (2021)** [13]	Nepal	Cadaver	16	-	6.25	28.12	56.25	18.75	-	-	-	-
**Tong H. et al. (2022)** [8]	China	Carotid DSA and color Doppler ultrasound	186	1.09	5.45	28.36	30.54	25.09	6.54	2.90	-	-
**Amarttayakong S. et al. (2024)** [14]	Thailand	CT Angio	86	4.65	5.81	44.19	14.53	27.33	1.74	1.74	-	-
**Manta M.D. et al. (2024)** [15]	Romania	CT Angio	147	1.02	7.48	27.21	26.19	25.51	5.1	7.14	0.34	-

A high carotid bifurcation presents numerous challenges that impact both surgical and endovascular management. Due to its proximity to the hypoglossal and glossopharyngeal nerves, there is an elevated risk of nerve injury during carotid endarterectomy, which may result in dysphagia, speech impairment, or Horner’s syndrome [2,18]. Due to being technically demanding, accessing a high carotid bifurcation often necessitates additional procedures such as styloidectomy or temporary jaw repositioning to enhance the exposure [20]. This anatomical variation also associates with a higher risk of ICA thrombosis, increasing the likelihood of postoperative stroke or transient ischemic attacks [18]. Intraoperative shunting is particularly challenging in these cases, sometimes leading to embolization or arterial dissection. Carotid artery stenting can also be complicated due to difficult catheter navigation and a higher risk of restenosis. Given these risks, preoperative imaging is paramount to determine the bifurcation level and select the most suitable revascularization strategy. In certain instances, carotid artery stenting may be preferred over carotid endarterectomy to minimize nerve injury and enhance outcomes. Additionally, patients with a high carotid bifurcation exhibit a greater propensity to develop postoperative hematomas, which, in severe cases, may even compromise the airway function [21,22].

From an embryological perspective, three primary theories have been proposed to elucidate the origin of high carotid bifurcation. These theories suggest that the ECA originates from the apex of the third aortic arch or directly from the dorsal aorta, while the ICA arises from the second aortic arch [2,23]. Any disruption in the timing of regression or elongation of the primitive maxillofacial trunk and the external carotid sprout can lead to unusual branching patterns [24]. Recognizing these variations is paramount in surgical and interventional planning, as they can significantly influence approaches in carotid surgeries, endovascular interventions, and head and neck oncological procedures.

A notable finding in the first case was the direct origin of the STA from the CCA on both sides. While the STA typically originates from the ECA, variations in its origin are not uncommon. A comprehensive review of 4,048 heminecks revealed that the STA arises from the CCA in approximately 20.28% of cases, with the majority originating from the ECA (51.74%) and the carotid bifurcation (24.61%) [25]. This variation holds significant clinical relevance, particularly in thyroid and carotid surgeries, where an unexpected STA origin can increase the risk of vascular injury, excessive bleeding, and potential damage to the external branch of the superior laryngeal nerve [25,26]. Variations such as the retropharyngeal course of STA have been reported, which are susceptible to damage in retropharyngeal abscesses and can lead to ischemia of the thyroid gland [27]. In endovascular procedures such as angiography or embolization of thyroid tumors, unrecognized STA anomalies can complicate catheter navigation and misinterpret the vascular anatomy. Furthermore, in cases of carotid artery disease, particularly during endarterectomy or stenting, an STA originating from the CCA may necessitate meticulous preoperative imaging to ensure optimal surgical planning.

In addition to the high carotid bifurcation and an anomalous origin of the STA, this case presented an unusual pentafurcation of the ECA at the carotid sinus. Typically, the ECA bifurcates into two terminal branches: the maxillary artery and the superficial temporal artery. Few studies have documented tri-ramification of CCA into STA, ECA and ICA. However, in the present study, five arteries arose directly from the ECA – the ascending pharyngeal, lingual, facial, and occipital arteries independently – while a common trunk gave rise to the posterior auricular, superficial temporal, and maxillary arteries. This differs from the previously reported cases where pentafurcation occurred deep to the angle of the mandible, with five distinct branches – ascending pharyngeal, facial, occipital, superficial temporal, and maxillary arteries – while the PAA originated from the superficial temporal artery [24]. The branching pattern was normal in Case two, whereas Case three shows that a high carotid bifurcation is not always associated with variation in the branching of ECA. Such a complex branching pattern at the carotid sinus poses significant challenges in procedures such as carotid endarterectomy and embolization, as it alters surgical landmarks and increases the risk of vascular injury.

## Conclusion

In conclusion, this case of bilateral high carotid bifurcation with pentafurcation of the external carotid artery represents a rare anatomical variation with substantial surgical and interventional implications. The presence of a STA arising from the CCA and the atypical pentafurcation emphasize the necessity for comprehensive preoperative imaging utilizing computed tomography angiography or Doppler ultrasound in patients undergoing interventions in the carotid, parotid, or upper cervical regions. Recognizing these anatomical complexities is indispensable for optimizing surgical planning, minimizing intraoperative risks, and ultimately improving patient outcomes.

## References

[1] Mirjalili SA, McFadden SL, Buckenham T, Stringer MD (2012). Vertebral levels of key landmarks in the neck. *Clin Anat*.

[2] Michalinos A, Chatzimarkos M, Arkadopoulos N, Safioleas M, Troupis T (2016). Anatomical Considerations on Surgical Anatomy of the Carotid Bifurcation. *Anat Res Int*.

[3] Rockley M, Ryan SE, Nagpal S (2020). Endarterectomy of carotid artery bifurcation in the setting of a persistent hypoglossal artery and anomalous collateral vascular supply. *J Vasc Surg Cases Innov Tech*.

[4] Standring S (2016). Gray’s anatomy: the anatomical basis of clinical practice.

[5] Anangwe D, Saidi H, Ogeng’o J, Awori KO (2008). Anatomical variations of the carotid arteries in adult Kenyans. *East Afr Med J*.

[6] Badhe P, Dabharde P, Kapadnis AS, Ravi Kiran D (2018). Anatomical Variation of Common Carotid Artery Bifurcation In Relation To Cervical Vertebra on CT Angiogram. *IOSR Journal of Dental and Medical Sciences (IOSR-JDMS)*.

[7] Radha K (2014). Bifurcation levels of the common carotid arteries-A cadaveric study in South Indian population. *Int J Anat Res*.

[8] Tong H, Sili Z, Xu S, Jie J, Jun B, Jianjin W, Liang W, Qingjun J, Lefeng Q (2022). [Evaluation of the level of carotid bifurcation and the morphology of extracranial internal carotid artery in patients with carotid stenosis by color doppler ultrasound and digital subtraction angiography]. *Natl Med J China*.

[9] Zümre Ö, Salbacak A, Çiçekcibaşi AE, Tuncer I, Seker M (2005). Investigation of the bifurcation level of the common carotid artery and variations of the branches of the external carotid artery in human fetuses. *Ann Anat*.

[10] Jitpun E, Wattanasen Y, Tirakotai W (2019). Do Asians have Higher Carotid Bifurcation? A Computed Tomographic Angiogram Study of the Common Carotid Artery Bifurcation and External Carotid Artery Branching Patterns. *Asian J Neurosurg*.

[11] Kurkcuoglu A, Aytekin C, Oktem H, Pelin C (2015). Morphological variation of carotid artery bifurcation level in digital angiography. *Folia Morphol (Warsz)*.

[12] Woldeyes Bahir D, Dh W (2014). Anatomical Variations of the Common Carotid Artery Bifurcations in Relation to the Cervical Vertebrae in Ethiopia 2014. *Anat Physiol*.

[13] Chalise U, Pradhan A, Lama CP, Dhungel S (2021). Bifurcation of Common Carotid Artery in Relation to Vertebral Level in Nepalese: A Cadaveric Study. *Nep Med Coll J*.

[14] Amarttayakong S, Amarttayakong P, Munkong W, Laup A, Chaiyamoon A, Suwannakhan A, Sangkhano S (2024). Is low carotid bifurcation determined by vertebral level always convenient for surgical approach?. *PLoS One*.

[15] Manta MD, Rusu MC, Hostiuc S, Tudose RC, Manta BA, Jianu AM (2024). The vertical topography of the carotid bifurcation–original study and review. *Surg Radiol Anat*.

[16] Gulsen S, Caner H, Altinors N (2009). An anatomical variant: Low-lying bifurcation of the common carotid artery, and its surgical implications in anterior cervical discectomy. *J Korean Neurosurg Soc*.

[17] McNamara JR, Fulton GJ, Manning BJ (2015). Three-dimensional Computed Tomographic Reconstruction of the Carotid Artery: Identifying High Bifurcation. *Eur J Vasc Endovasc Surg*.

[18] Kazantsev AN, Korotkikh AV, Lider RY (2023). An alternative anatomical classification for carotid bifurcation and impact on outcome of carotid endarterectomy: a multicenter study. *Cardiothorac Surg*.

[19] Keshelava G, Kovziridze D, Robakidze Z (2023). 180-degre carotid bifurcation with an internal carotid artery aneurysm. *Surg Radiol Anat*.

[20] Conte Neto N, Gonçalves TT, Louis C, Ikikame J, Góes Junior AMO (2022). Surgical access to the distal cervical segment of the internal carotid artery and to a high carotid bifurcation–integrative literature review and protocol proposal. *J Vasc Bras*.

[21] Galyfos G, Chamzin A, Sigala F, Filis K (2023). Postcarotid Endarterectomy Hematoma Induced Arrhythmia: Report of a Rare Case. *Case Rep Vasc Med*.

[22] Comerota AJ, Difiore R, Tzilinis A, Chahwan S (2012). Cervical hematoma following carotid endarterectomy is morbid and preventable: A 12-year case-controlled review. *Vasc Endovascular Surg*.

[23] Brown CR, Brown ND, Brown MR, Kurt G, Özer A (2024). Surgical Considerations and Strategies for Anatomically Variant and Diseased Carotid Arteries. *Carotid Arteries - Function, Pathology and Treatment* IntechOpen.

[24] Moraru L, Rusu MC, Popescu ŞA (2021). True terminal pentafurcation of the external carotid artery and terminal trifurcation of the contralateral one, occipitoauricular trunk, retropharyngeal internal carotid artery. *Surg Radiol Anat*.

[25] Tzortzis AS, Antonopoulos I, Pechlivanidou E, Chrysikos D, Pappas N, Troupis T (2023). Anatomical variations of the superior thyroid artery: A systematic review. *Morphologie*.

[26] Dessie MA (2018). Variations of the origin of superior thyroid artery and its relationship with the external branch of superior laryngeal nerve. *PLoS One*.

[27] Calotă RN, Rusu MC, Dumitru CC, Moraru L, Tudose RC (2025). Retropharyngeal course of the superior thyroid artery–a novel finding. *Surg Radiol Anat*.

